# The Effect of Mycorrhization with Fungi of Different Efficiency on the Root Metabolome of *Medicago lupulina* Within Development

**DOI:** 10.3390/ijms27177751

**Published:** 2026-08-29

**Authors:** Andrey P. Yurkov, Roman K. Puzanskiy, Ekaterina M. Bogdanova, Alexey A. Kryukov, Tatyana R. Vavulina, Angelina I. Belyaeva, Anastasia I. Kosulnikova, Yuri V. Kosulnikov, Yuri V. Laktionov, Vladislav V. Yemelyanov, Alexey L. Shavarda, Maria F. Shishova

**Affiliations:** 1 Laboratory of Ecology of Symbiotic and Associative Rhizobacteria, All-Russia Research Institute for Agricultural Microbiology, Pushkin, 196608 St. Petersburg, Russia; aa.krukov@arriam.ru (A.A.K.); t.kudryashova@arriam.ru (T.R.V.); ai.belyaeva@arriam.ru (A.I.B.); a.kovalchuk@arriam.ru (A.I.K.); yv.kosulnikov@arriam.ru (Y.V.K.); laktionov@arriam.ru (Y.V.L.); 2 Faculty of Biology, St. Petersburg State University, 199034 St. Petersburg, Russia; puzansky@yandex.ru (R.K.P.); bogdanova.ekaterina15@gmail.com (E.M.B.); v.yemelyanov@spbu.ru (V.V.Y.); m.shishova@spbu.ru (M.F.S.); 3 Laboratory of Analytical Phytochemistry, Komarov Botanical Institute of the Russian Academy of Sciences, 197022 St. Petersburg, Russia; a.shavarda@spbu.ru; 4 Graduate School of Biotechnology and Food Science, Peter the Great St. Petersburg Polytechnic University, 194021 St. Petersburg, Russia; 5 Center for Molecular and Cell Technologies, St. Petersburg State University, 199034 St. Petersburg, Russia

**Keywords:** mycorrhizal growth response, effective symbiosis, ineffective symbiosis, arbuscular mycorrhiza, metabolic profile, GC-MS, phosphorus deficiency, biochemical networks, *Medicago lupulina*, *Rhizophagus irregularis*

## Abstract

The mechanisms underlying the symbiotic efficiency of arbuscular mycorrhizal (AM) fungi are actively debated, but comparative metabolomic studies with fungi of contrasting efficiency are scarce. This study aimed to evaluate the influence of effective (*Rhizophagus irregularis* RCAM00320) and ineffective (*Glomus* sp. 129.1Te) AM fungal strains on the root metabolome of the responsive *Medicago lupulina* line MlS-1 at two vegetative and two reproductive stages. Using GC-MS, over 150 metabolites (amino acids, carboxylic and fatty acids, sugars, etc.) were annotated. Effective AM symbiosis was associated with increased levels of phosphoric acid, trehalose, and free fatty acids 16:1, as well as a decreased pool of tricarboxylic acid cycle intermediates (citrate, malate, succinate) and a reduction in γ-aminobutyric acid from the branching stage to fruiting. The comparison revealed that the branching initiation stage, characterized by low arbuscule abundance in ineffective treatment, is likely a main critical metabolic transition determining symbiosis efficiency. Novel metabolic markers of effective AM were identified. Network analysis revealed a divergence of amino acid and fatty acid clusters under effective mycorrhization, whereas under ineffective mycorrhization these clusters were less separated and closely linked in the control. Thus, inoculation with strains of contrasting efficiency generates distinct phenotypes, with effective AM inducing the most pronounced metabolome rearrangements in development.

## 1. Introduction

Arbuscular mycorrhizal (AM) fungi from the monophyletic phylum Glomeromycota [1,2] are unique symbionts that can greatly increase the absorptive surface area of roots and provide up to 90–99% of inorganic phosphorus (Pi) uptake by plants through specialized fungal transporters [3,4,5].

A substantial body of evidence indicates that AM symbiosis enhances host plant tolerance to various abiotic and biotic stresses [4,6,7,8,9,10,11], profoundly affecting adaptive processes at the transcriptomic, proteomic levels, and especially the metabolomic level [12,13,14]. For example, inoculation of *Puccinellia tenuiflora* with *Rhizophagus intraradices* significantly increased amino acid, organic acid, flavonoid, and sterol content, improving osmotic regulation and maintaining cell membrane stability under alkaline stress [6]. In soybean, co-inoculation with a mixture of AM fungi (*Acaulospora laevis*, *Septoglomus deserticola*, and *R. irregularis*) and *Bacillus amyloliquefaciens* enhanced phenolic, flavonoid, soluble sugar, lipid, protein, and oil content under drought conditions [9]. Colonization of *Poncirus trifoliate* by *R. intraradices* was shown to reduce the content of several terpenoids under drought stress (except *β*-caryophyllene), suggesting a potential increase in volatile organic compounds to initiate plant defense responses [10]. Recently, the effect of *R. irregularis* on the root metabolome of *M. lupulina* was analyzed under different phosphorus regimes (low and optimal Pi availability; [11]), revealing 14 key metabolites involved in effective AM symbiosis development (manifested in a significant increase in the biomass of AM plants compared to non-AM control) under varying substrate phosphorus levels. A proteomic study of *Sorghum bicolor* mycorrhized with a consortium of AM fungi (*Septoglomus constrictum*, *Funneliformis geosporum*, *Rhizoglomus fasciculatum*, *Glomus toruosum*, *Gigaspora margarita*, and *Acaulospora scrobiculata*) identified three proteins involved in the adaptation of mycorrhizal plants to drought [8]. A large-scale evaluation of the effect of *Glomus versiforme* on the growth and development of fourteen bioenergy accessions of *S. bicolor* revealed variation in AM symbiosis efficiency and identified 55 differentially expressed genes among *S. bicolor* genotypes, with some genes differentially expressed during symbiosis in at least one genotype [12]. Recent transcriptomic studies have shown that plant responses to AM depend on the genotype. Virág et al. [15] demonstrated that colonization by *F. mosseae* triggers genotype-dependent transcriptional programs in maize roots under drought conditions, with the extent of transcriptional reprogramming differing markedly between tolerant and susceptible inbred lines. Similarly, Virág et al. [16] showed that AM-mediated drought adaptation involves genotype-dependent modulation of photosynthetic, hormonal, and root developmental processes, indicating that the symbiotic effect is not universal but depends on the host plant genotype. These findings support the view that AM efficiency is governed by molecular interactions specific to the plant-fungus pair in the plant-microbe system (plant line and fungal strain). This underscores the need for comparative analyses using PMS contrasting in symbiotic efficiency.

Despite the considerable number of studies on the adaptive responses of AM plants to stressors, the molecular and metabolic mechanisms determining the differential efficiency of AM symbiosis in the absence of stress remain poorly understood [5]. This is partly due to the difficulty of selecting suitable model plant-fungus pairs and the obligate biotrophic status of the mycosymbiont. Notably, some model plant species are less appropriate to study metabolic and transcriptomic rearrangements during mycorrhization. For instance, in *Pisum sativum* under high Pi, even the addition of exogenous strigolactones did not intensify root colonization, indicating the existence of additional regulatory mechanisms affecting hyphopodium differentiation [14]. Model plants that do not exhibit a substantial growth response to AM development with *R. irregularis* include several accessions of *Plantago lanceolata*, *Plantago major*, *Veronica chamaedrys*, *Poa annua*, and *Medicago truncatula*, which are characterized by a lack of mycorrhizal growth response (MGR), forming ineffective AM [17]. Therefore, to elucidate the mechanisms underlying effective AM development, a comparative analysis of specially selected effective and ineffective model symbiont pairs with contrasting (differential) mycorrhizal responses is warranted.

It is known that some AM fungi can form more effective symbioses with particular plant species. For example, in *Juglans regia*, effective symbiosis (with significant MGR) has been demonstrated with various AM fungi, including *Acaulospora scrobiculata*, *Diversispora spurca*, *Glomus etunicatum*, *F. mosseae*, *G. versiforme*, and others [18], with *D. spurca* being the dominant and most effective species [19]. Analysis of the plant-microbe system (PMS) formed by *Solanum lycopersicum* cultivar Moneymaker with different AM fungi (*F. mosseae* strain BEG12, *Rhizoglomus irregulare* strain DAOM 197198, formerly *Rhizophagus irregularis*, and *Claroideoglomus etunicatum* strain EEZ163) showed that MGR under non-stress conditions did not differ among inoculation treatments [20]. Even under drought or salinity stress, AM efficiency showed the absence of common trends across inoculation treatments. Nevertheless, the authors detected significant metabolomic reprogramming in mycorrhizal roots of plants grown under various drought or salinity stress conditions [20]. Inoculation with *Claroideoglomus etunicatum* induced more substantial metabolic rearrangements than mycorrhization with *F. mosseae* or *R. irregularis*. Despite these findings, these studies do not allow assessment of the intensity and directionality of metabolic rearrangements depending on the specific plant-strain pair. Data on the long-term dynamics of the metabolome during symbiosis development are limited to a small number of plant species, including *Stevia rebaudiana* [21], *Pisum sativum* [22,23], *Medicago lupulina* [24], and *Solanum lycopersicum* [25,26]. Moreover, these studies do not directly compare metabolomic changes depending on the efficiency of the specific symbiont pair.

The first direct comparison of metabolism during the formation of effective and ineffective AM symbiosis at 30 DAS (without studying long-term dynamics) was performed on sorghum [27]. Two plant lines—*Sorghum caudatum* accession PI-297130 and *S. bicolor* accession PI-562730—were analyzed in symbiosis with the effective mutualistic *Rhizophagus irregularis* isolate FL208A and *Gigaspora gigantea* isolate MN922A, which exhibits ineffective fungal properties (MGR with negative values). Among the 49 primary metabolites analyzed, no specific markers, unequivocally characteristic of effective AM symbiosis, were identified. Nevertheless, the authors admitted that under symbiosis with multiple AM fungi, the host plant can redistribute resources in favor of mutualistic fungal partners. Interestingly, in other studies comparing the efficiency of different AM fungal species (*Glomus versiforme*, *R. irregularis*, *Claroideoglomus claroideum*, *Gigaspora gigantea*), *G. gigantea* consistently formed symbioses with the lowest MGR when inoculating 18 *S. bicolor* lines [12]. That can also indicate differences in metabolomic rearrangements depending on the efficiency of the interaction between the host plant and the AM fungus.

These data underscore the necessity of a comparative analysis of effective and ineffective symbiotic relationships. The highly responsive line MlS-1 of *Medicago lupulina*, which exhibits a consistently high MGR with the effective strain RCAM00320 of *Rhizophagus irregularis* and no MGR with the ineffective strain 129.1Te of *Glomus* sp., is a suitable model system [11,28].

Based on our previous findings on metabolic rearrangements associated with effective AM [11,24], we hypothesize that a comparative analysis of effective and ineffective symbiotic pairs will allow the identification of metabolites specific to effective AM. Accordingly, this study aims to elucidate differences in metabolic rearrangements under the development of effective and ineffective arbuscular mycorrhizal symbiosis.

## 2. Results

### 2.1. Changes in Productivity and Arbuscule Abundance in the Roots of M. lupulina as a Functional Criteria of AM Fungal Strain Efficiency

The effect of inoculation with effective (RCAM00320) and ineffective (129.1Te) AM fungal strains on plant productivity and root mycorrhization of *M. lupulina* line MlS-1 was assessed at four key developmental stages of the host plant: 2nd leaf (21 days after sowing, DAS), branching initiation (35 DAS), flowering initiation (48 DAS), and fruiting initiation (54 DAS). Plants of *M. lupulina* line MlS-1 differed in their development upon inoculation with effective (strain RCAM00320) and ineffective (strain 129.1Te) AM fungi when grown in a sterilized soil-sand mixture (Figure S1).

Inoculation with the effective strain RCAM00320 resulted in AM development with high symbiotic efficiency, as measured by MGR based on fresh weight per plant at all chosen stages of development (Figure S1A). The highest symbiotic efficiency was observed at the branching initiation and flowering initiation stages (+181.8–203.9%). The ineffective strain 129.1Te did not exhibit substantial symbiotic efficiency, although a slight increase in plant weight compared to the control was observed at the reproductive stages (48 and 54 DAS; +16.2–19.4%). At the vegetative stages (2nd leaf, 21 DAS, and branching initiation, 35 DAS), significant differences in the root fresh weight to shoot fresh weight ratio (RFW:SFW) were observed between the non-inoculated control and the mycorrhizal treatments (both effective and ineffective AM fungi; Figure S1B). Plant development with and without mycorrhiza under low available phosphorus conditions differed: in an attempt to adapt to environmental conditions, non-AM plants formed a more extensive root system for the same shoot weight compared to AM plants. However, for the effective and ineffective AM symbiosis treatments, the RFW:SFW ratio did not differ significantly throughout host plant development. At the same time, in the later stages (48 and 54 DAS), root development relative to shoot development in the non-AM treatment slows down, and no significant differences between the mycorrhizal treatments were found at flowering initiation and fruiting initiation.

The primary symbiotic structures of AM that facilitate metabolite exchange are arbuscules (so-called “symbiosomes”). Analysis of mycorrhization based on arbuscule abundance in roots (*A*, %) revealed significantly lower arbuscule abundance in the ineffective AM fungal inoculation treatment compared to the effective fungal inoculation (Figure S1C). During vegetative development (21 and 35 DAS), mycorrhization by the ineffective AM fungus was extremely low (*A* = 0.2–1.0%), and increased markedly at the reproductive stages to *A* = 9.1–17.7%. The development of effective AM symbiosis in the *M. lupulina* line MlS-1 with *R. irregularis* strain RCAM00320 was active, with arbuscule abundance, *A* elevated from 14.0% at the 2nd leaf stage to 34.4% by the fruiting initiation.

### 2.2. General Characteristics of Root Metabolite Profiles of M. lupulina Line MlS-1 upon Mycorrhization with Effective and Ineffective AM Fungi

In the obtained root metabolite profiles of *M. lupulina*, more than 250 mass spectra were identified, of which over 150 were annotated (Table S1). A set of metabolites whose levels differed between effective and ineffective mycorrhization in at least three out of four developmental stages is presented in Table S2 (VIP > 1). For more than 100 of the annotated mass spectra, exact identities were established (using library databases), while for the remainder, the compound class was determined. Sugars and their derivatives, including pentoses, hexoses, and glycosides, were the most abundant. A total of 26 amino acids, 20 carboxylic acids (including energy metabolism intermediates), 27 free fatty acids (FFAs) and their derivatives, as well as nitrogenous bases, sterols, phenolic compounds, and others, were detected.

Figure S2 presents a heatmap of the average metabolite content in *M. lupulina* roots. Metabolites were clustered using Ward’s method with Pearson distance. A clear trend toward grouping of metabolically related compounds was observed. Clusters included tricarboxylic acid (TCA) cycle intermediates, and amino acids were distinguished. The obtained data indicated that the regulation of the TCA cycle and amino acid metabolism is under tight control linked to host-plant and mycorrhiza development.

**Figure 1:**
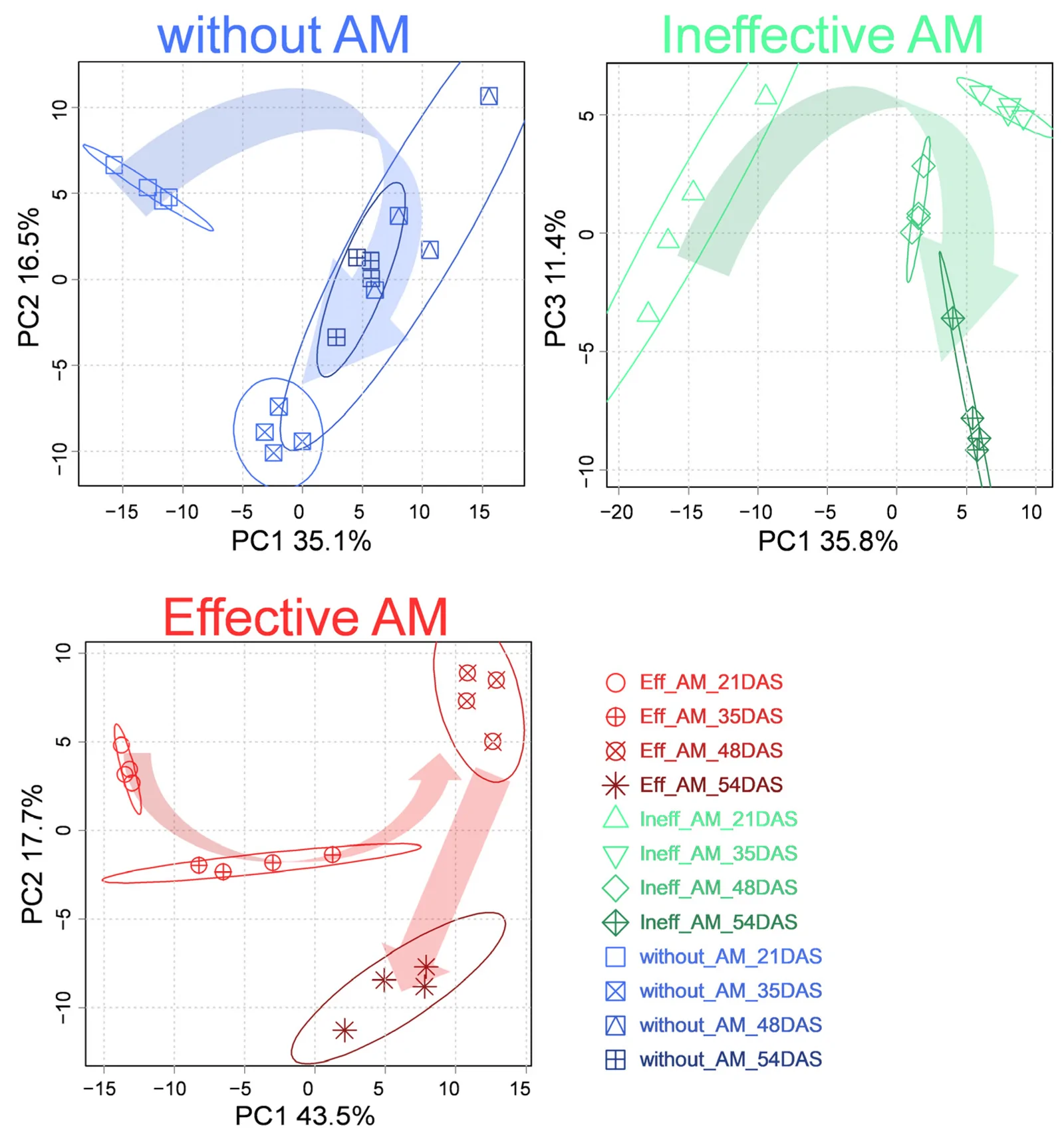
Mycorrhization effects trajectory of metabolome temporal alteration. Ordination of metabolite profiles in PCA scores. % is the percentage of variance related to the principal component (PC), ellipses are 90% CI. 21 DAS, 35 DAS, 48 DAS, 54 DAS—21, 35, 48, and 54 days after sowing and inoculation, respectively. “without_AM”—control without AM fungal inoculation; “Ineff_AM”—ineffective AM, inoculation with ineffective *Glomus* sp. strain 129.1Te; “Eff_AM”—effective AM, inoculation with effective *Rhizophagus irregularis* strain RCAM00320.

To identify and visualize similarities and differences in the root metabolomic profiles of *M. lupulina* at different stages, the profiles were projected into lower-dimensional spaces using Principal Component Analysis (PCA). In Figure 1, the metabolite profiles of roots from plants with different mycorrhization statuses are shown in the space of the first two principal components (PCs). In all three treatments (non-AM and two mycorrhizal treatments), the metabolome changed substantially during plant development. In all treatments, differences between the initial and final developmental time points were associated with PC1, which explained 35%, 37%, and 42% of the variance for the non-mycorrhizal (“without_AM”), ineffective AM (129.1Te—“Ineff_AM”), and effective AM (RCAM00320—“Eff_AM”) treatments, respectively. Moreover, the developmental trajectory was non-linear and differed markedly among the roots of plants from the three variants. In the control (without AM), a sharp transition occurred between 21 DAS and 35 DAS, with differences between 21- and 35-day-old plants attributed not only to PC1 but also to PC2 (17%). Differences at later stages were less pronounced. In the ineffective AM treatment (129.1Te), a similar transition was observed but was associated only with PC1. For roots of plants with ineffective AM, differences at later stages were also weakly expressed along PC1, whereas roots of 48-day-old plants differed along PC2 (24%). In the case of effective AM formation (RCAM00320), such a large transition between 21 DAS and 35 DAS was not observed. More substantial changes occurred between 35 DAS and 48 DAS (during the transition from branching to flowering). As in the previous cases, differences at later stages were less pronounced along PC1, whereas roots of 54-day-old plants showed substantial differences along PC2 (18%).

**Figure 2:**
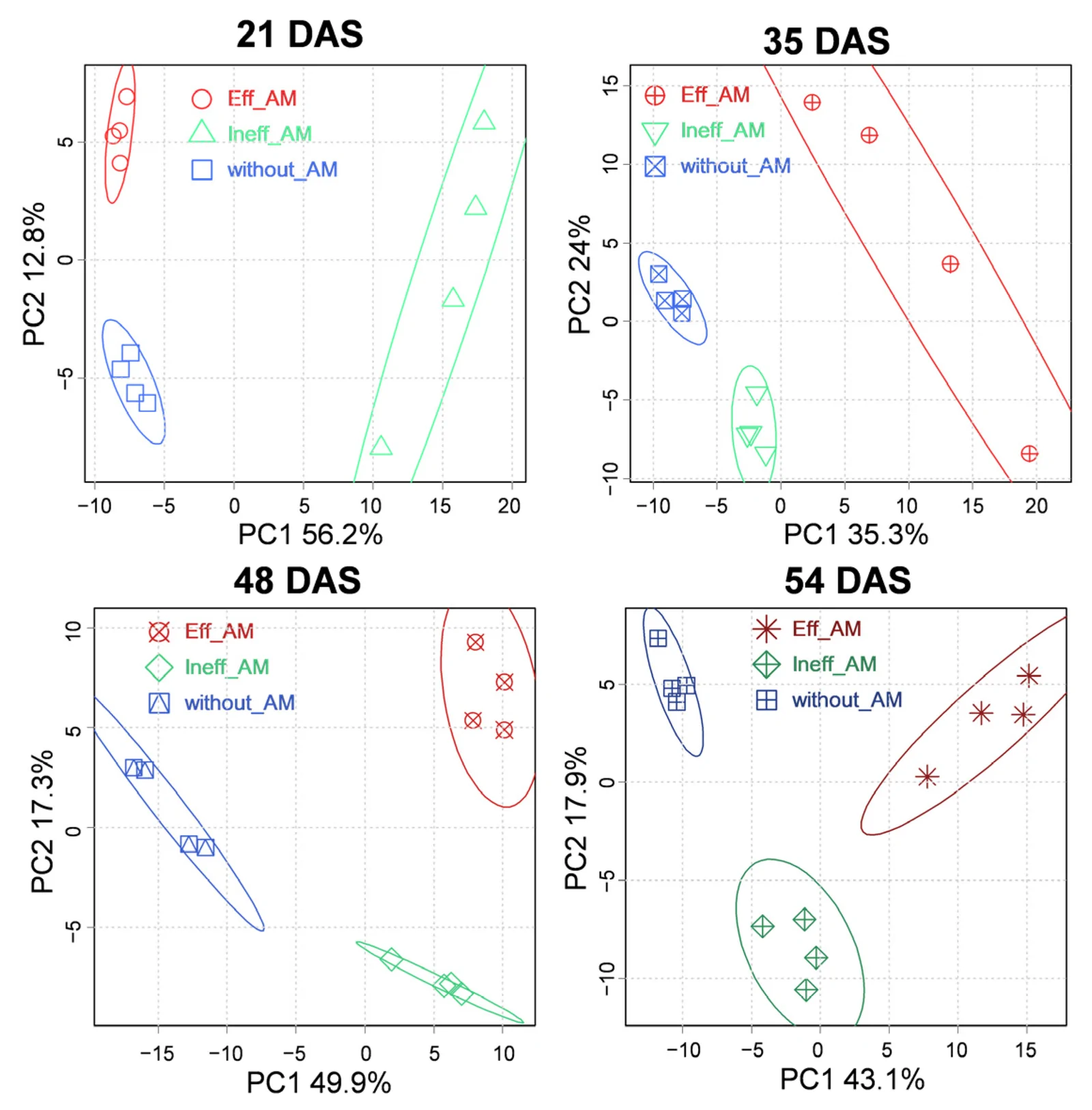
PCA score plots for every time point. %—percentage of variance related to the principal component (PC), ellipses—90% CI. For explanations, see Figure 1 and Figure S2.

Since host plant development exerted a substantial influence on the root metabolome, the effect of mycorrhization was analyzed separately for each age group. In Figure 2, the root profiles of plants at four stages are shown in the space of two PCs. In all cases, samples grouped according to mycorrhization status. It should be noted that at all stages except the first (21 DAS), the “without_AM” (control) and effective inoculation (RCAM00320) treatments were maximally separated, while the ineffective mycorrhization treatment (129.1Te) occupied an intermediate position.

### 2.3. Effect of Mycorrhization on the Root Metabolome at 21 DAS

To identify differences between the non-inoculated control and the two types of mycorrhiza (effective and ineffective), OPLS-DA classification was performed. The models included one predictive and one orthogonal component. In the effective AM treatment (strain RCAM00320), the predictive component accounted for 44% of the variance in metabolite content. In the 129.1Te treatment (ineffective AM), this value was highest (over 58%).

**Figure 3:**
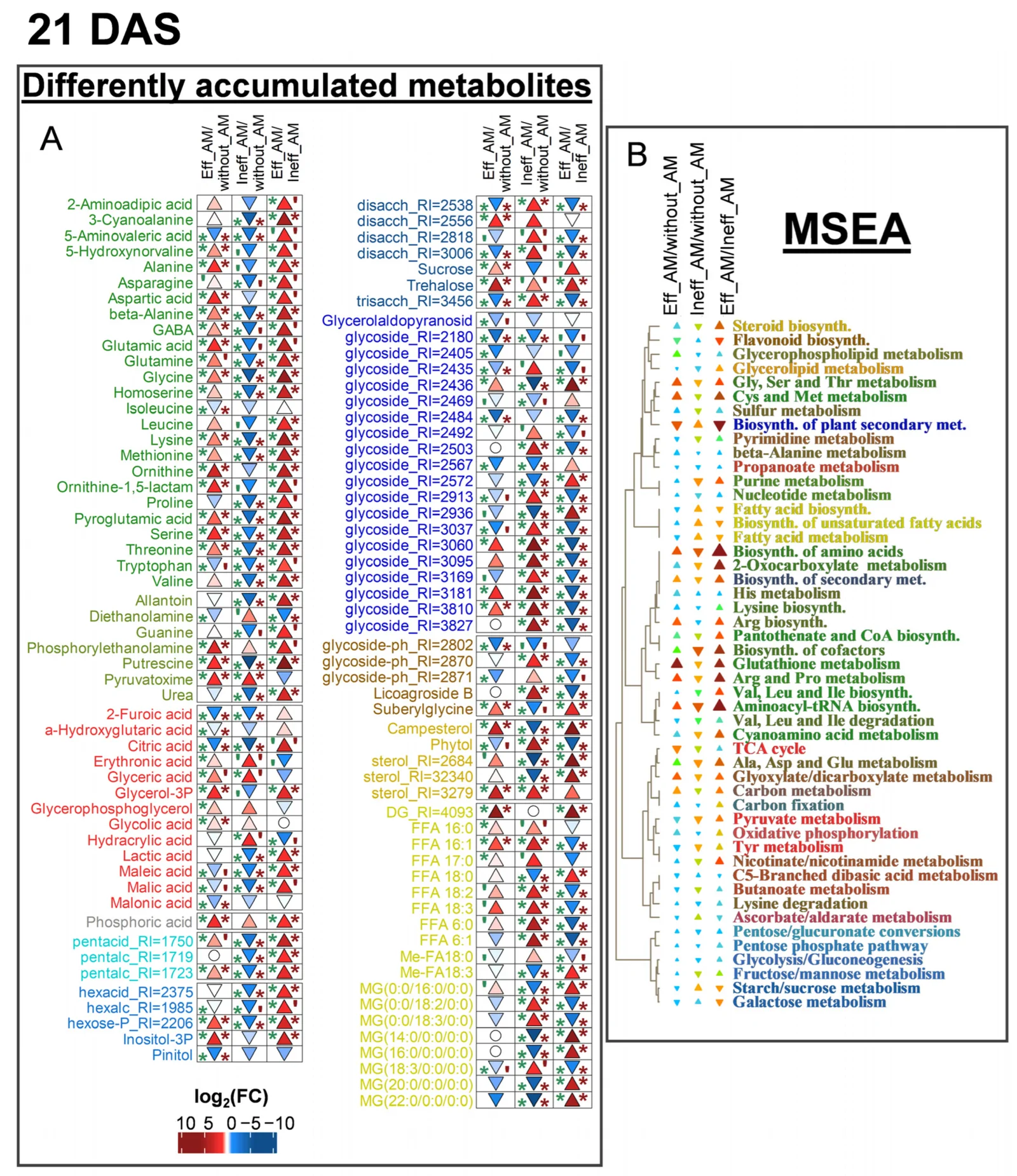
Metabolites differently accumulated in roots of 21-day plants. (**A**). Heatmap of Fold Changes (log_2_(FC)). Green asterisks at the left—VIP > 1, single quotation mark = 1 > VIP > 0.8. Red asterisks at the right—FDR < 0.05, quotation 0.05 < FDR < 0.1. (**B**). Metabolite Sets Enrichment Analysis (MSEA) based on ranking by loadings from OPLS-DA classification. “without_AM”—control without AM fungal inoculation; “Ineff_AM”—ineffective AM, inoculation with ineffective *Glomus* sp. strain 129.1Te; “Eff_AM”—effective AM, inoculation with effective *Rhizophagus irregularis* strain RCAM00320. The colors of the names of differentially accumulated metabolites are as follows: green—amino acids and derivatives; olive—other nitrogen-containing compounds; red—organic acids and alcohols; gray—inorganic phosphate; light blue—pentoses; blue—hexoses; dodger blue—oligosaccharides; dark blue—glycosides; brown—secondary metabolites; orange—sterols and terpenes; yellow—fatty acids and derivatives. Abbreviations: RI—retention index; pentalc, hexalc—sugar alcohols; pentacid, hexacid—sugar acids; FFA—free fatty acid; MG—monoacylglycerol; Me—methyl ether; glycoside-ph—glycosides with aromatic groups; disacch, trisacch—di- and trisaccharides.

Differentially accumulated metabolites (DAMs) belonged to various compound classes (Figure 3A). Effective mycorrhiza was associated with reduced accumulation of major TCA cycle intermediates, such as citrate, malate, and some other carboxylates. Concurrently, accumulation of amino acids and nitrogen-containing compounds increased. Additionally, accumulation of C3 compounds increased. In the effective AM treatment (RCAM00320), phosphorus and phosphorylated metabolite levels were elevated. Sucrose content was higher, but an unusual decrease in glycoside accumulation was observed. Enhanced amino acid metabolism against a background of TCA cycle repression was confirmed by MSEA (Figure 3B).

In both mycorrhizal treatments (by effective and ineffective AM fungi), repression of TCA cycle intermediate accumulation was observed, whereas accumulation of amino acids and nitrogen-containing metabolites decreased. Compared to the non-AM control, roots of plants with ineffective AM accumulated more FFAs, especially C16, but in the case of monoacylglycerols (MAGs), differences were bidirectional. Interestingly, in contrast to effective AM, the ineffective AM treatment exhibited increased glycoside accumulation. Comparison of effective and ineffective AM showed that effective symbiosis was associated with greater accumulation of TCA cycle intermediates, carboxylates, and amino acids. Furthermore, a trend toward greater acylglycerol accumulation was observed in the effective AM treatment, whereas FFAs accumulated more in the ineffective AM treatment.

### 2.4. Effect of Mycorrhization on the Root Metabolome at 35 DAS

OPLS-DA classification showed that in the comparison of the “without_AM” treatment with effective AM, the predictive component accounted for 43% of the variance in metabolite content. In the ineffective AM treatment, these values were similar (the predictive component also accounted for 38% of the variance). Thus, after 35 DAS, the effect of both types of mycorrhization was similar. Differences between plants with effective and ineffective AM were also moderate, with the predictive component of the OPLS-DA model accounting for 38% of variance.

**Figure 4:**
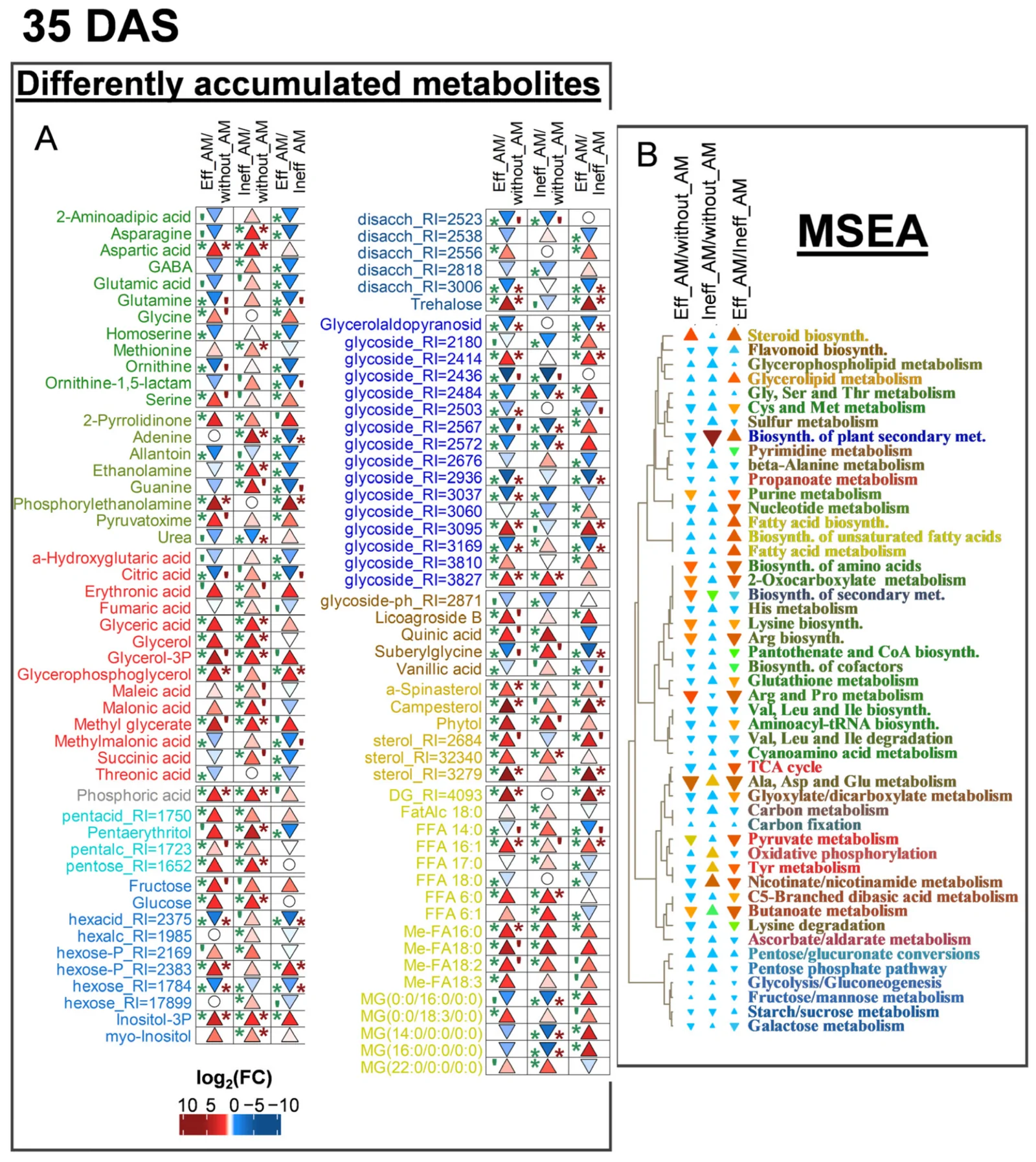
Metabolites differently accumulated in roots of 35-day plants. (**A**). heatmap of Fold Changes (log_2_(FC)). Green asterisks at the left—VIP > 1, single quotation mark = 1 > VIP > 0.8. Red asterisks at the right—FDR < 0.05, quotation 0.05 < FDR < 0.1. (**B**). Metabolite Sets Enrichment Analysis (MSEA) based on ranking by loadings from OPLS-DA classification. “without_AM”—control without AM fungal inoculation; “Ineff_AM”—ineffective AM, inoculation with ineffective *Glomus* sp. strain 129.1Te; “Eff_AM”—effective AM, inoculation with effective *Rhizophagus irregularis* strain RCAM00320. For explanations, see Figure 3.

Effective mycorrhiza repressed TCA cycle intermediate accumulation less markedly than at 21 DAS (Figure 4). In contrast to 21-day-old plants, at 35 DAS, the suppression of amino acid metabolism was observed, accompanied by an increase in the pool of metabolites related to lipid metabolism. As earlier, in the effective AM treatment, phosphorus and phosphorylated metabolite levels were higher.

In the ineffective AM treatment, decreased MAG accumulation was observed alongside increased FFA levels. Additionally, several glycosides decreased while monosaccharides, including glucose, increased. An interesting finding was the increased accumulation of some carboxylates related to oxidative phosphorylation, as well as a trend toward amino acid accumulation. As in the case of effective AM, the ineffective AM treatment also showed higher phosphorus and phosphorylated metabolite levels.

Comparison of effective and ineffective AM treatments showed that effective symbiosis was associated with greater accumulation of compounds related to glycerolipid, fatty acid, and sterol metabolism. By contrast, roots of plants with ineffective mycorrhiza exhibited increased accumulation of TCA cycle intermediates and amino acids (Figure 4).

### 2.5. Effect of Mycorrhization on the Root Metabolome at 48 DAS

**Figure 5:**
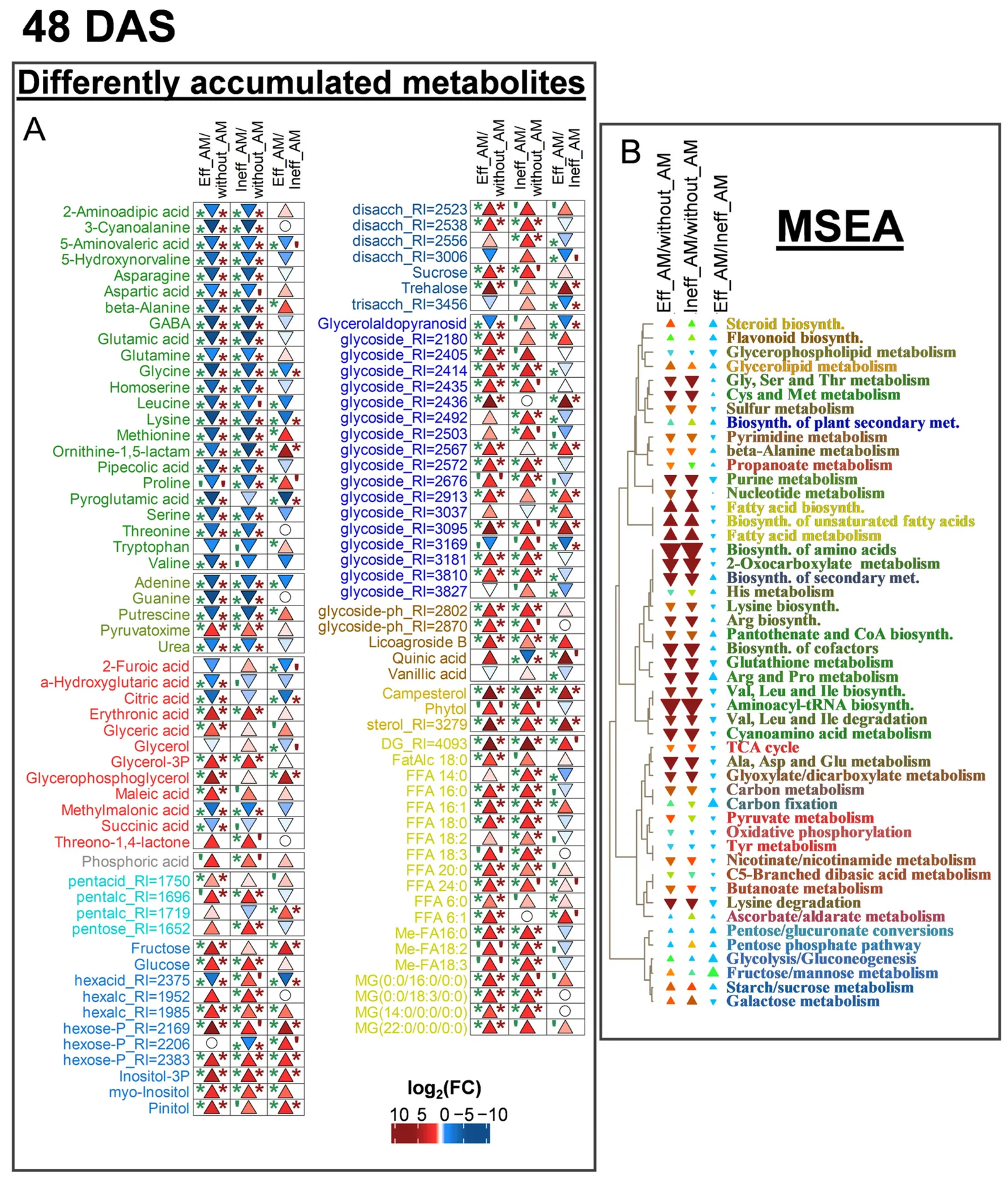
Metabolites differently accumulated in roots of 48-day plants. (**A**). heatmap of Fold Changes (log_2_(FC)). Green asterisks at the left—VIP > 1, single quotation mark = 1 > VIP > 0.8. Red asterisks at the right—FDR < 0.05, quotation 0.05 < FDR < 0.1. (**B**). Metabolite Sets Enrichment Analysis (MSEA) based on ranking by loadings from OPLS-DA classification. “without_AM”—control without AM fungal inoculation; “Ineff_AM”—ineffective AM, inoculation with ineffective *Glomus* sp. strain 129.1Te; “Eff_AM”—effective AM, inoculation with effective *Rhizophagus irregularis* strain RCAM00320. For explanations, see Figure 3.

OPLS-DA classification showed that in the comparison of the non-AM control with effective AM, the predictive component accounted for 61% of the variance in metabolite content. Thus, the effect of effective symbiosis was most pronounced at this stage. In the ineffective AM treatment, this value was 52% of the variance. As can be seen (Figure 2, Figure 5 and Figure S1A,C), at 48 DAS, the effect of mycorrhiza was more pronounced. In the comparison of effective and ineffective AM, the predictive component explained only 35% of the variance.

Effective mycorrhiza significantly suppressed the accumulation of TCA cycle intermediates, amino acids, and nitrogen-containing compounds, as confirmed by MSEA (Figure 5). An exception was 2-ketoglutarate, whose level was elevated. As before, roots of plants with effective AM accumulated more sugars and glycosides, including glucose, fructose, myo-inositol, and sucrose. They also accumulated more metabolites related to lipid metabolism. In terms of phosphorus and phosphorylated metabolites content, plants with effective AM outperformed the non-AM control, but this effect became less pronounced. At this stage, the effect of ineffective mycorrhiza became similar to that of effective AM. The few differences between effective and ineffective AM consisted of patterns of accumulation of a small number of lipid metabolism intermediates (campesterol, sterol RI = 3279), amino acids (beta-alanine, proline, putrescine), and sugars (trehalose, fructose, and some hexoses).

### 2.6. Effect of Mycorrhization on the Root Metabolome at 54 DAS

OPLS-DA classification showed that in the comparison of the non-AM control with the effective AM treatment, the predictive component accounted for 58% of the variance in metabolite content. In the ineffective AM treatment, this value was 41% of the variance. In the comparison of effective and ineffective AM, the predictive component explained only 43% of the variance.

**Figure 6:**
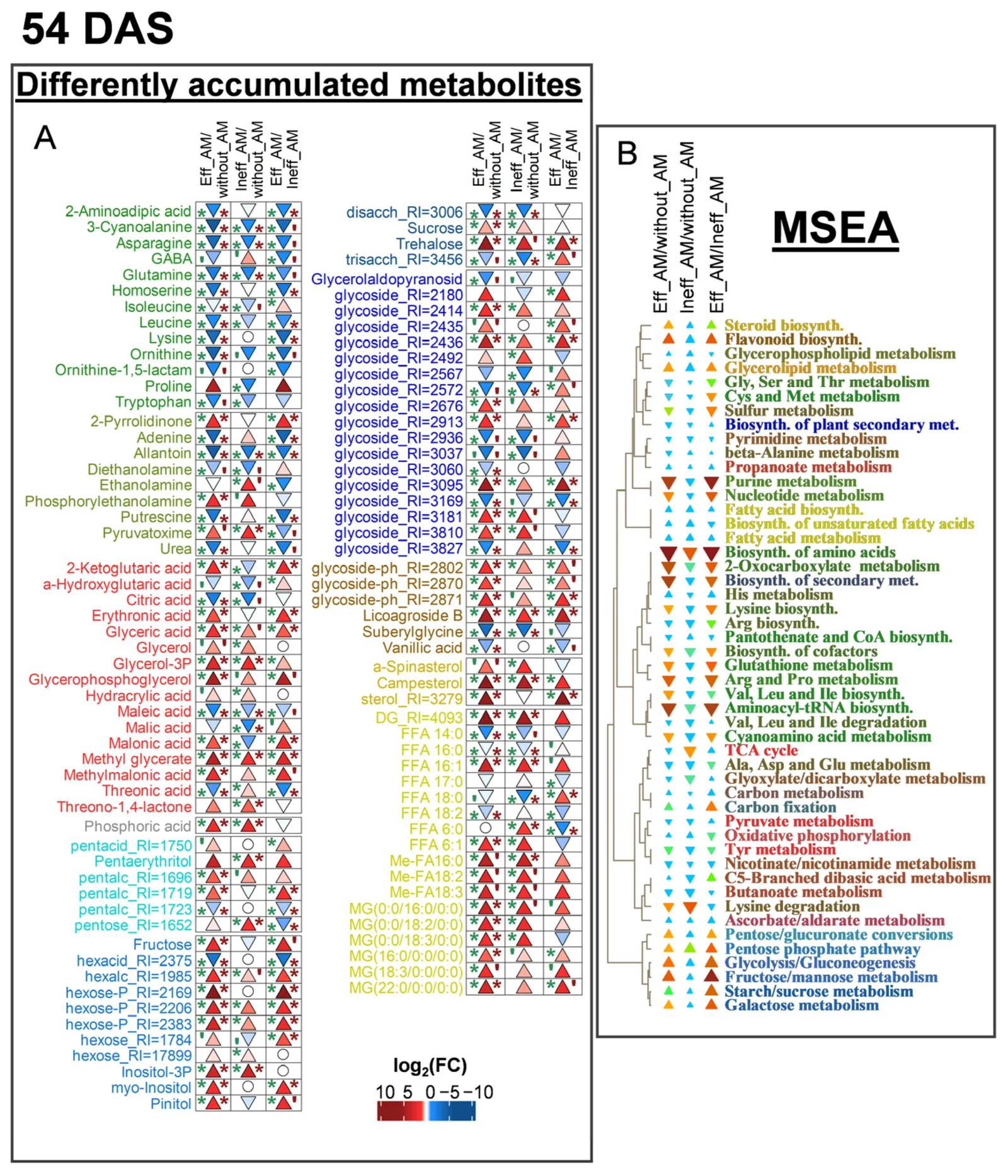
Metabolites differently accumulated in roots of 54-day plants. (**A**). heatmap of Fold Changes (log_2_(FC)). Green asterisks at the left—VIP > 1, single quotation mark = 1 > VIP > 0.8. Red asterisks at the right—FDR < 0.05, quotation 0.05 < FDR < 0.1. (**B**). Metabolite Sets Enrichment Analysis (MSEA) based on ranking by loadings from OPLS-DA classification. “without_AM”—control without AM fungal inoculation; “Ineff_AM”—ineffective AM, inoculation with ineffective *Glomus* sp. strain 129.1Te; “Eff_AM”—effective AM, inoculation with effective *Rhizophagus irregularis* strain RCAM00320. For explanations, see Figure 3.

Both effective and ineffective AM suppressed the accumulation of TCA cycle intermediates, pyruvate, and amino acids (Figure 6). However, in the effective mycorrhiza treatment, only citrate accumulation was significantly suppressed, while 2-ketoglutarate levels were elevated, as at the previous developmental stage. AM stimulated the accumulation of sterols and glycerolipid metabolism metabolites, with these effects being more pronounced in the effective AM treatment. Both inoculation treatments (effective and ineffective AM fungi) promoted the accumulation of sucrose and some other sugars. The stimulating effect on carbohydrate metabolism was more pronounced in plants with effective AM.

### 2.7. Mapping of Metabolites Based on Strong Correlations

**Figure 7:**
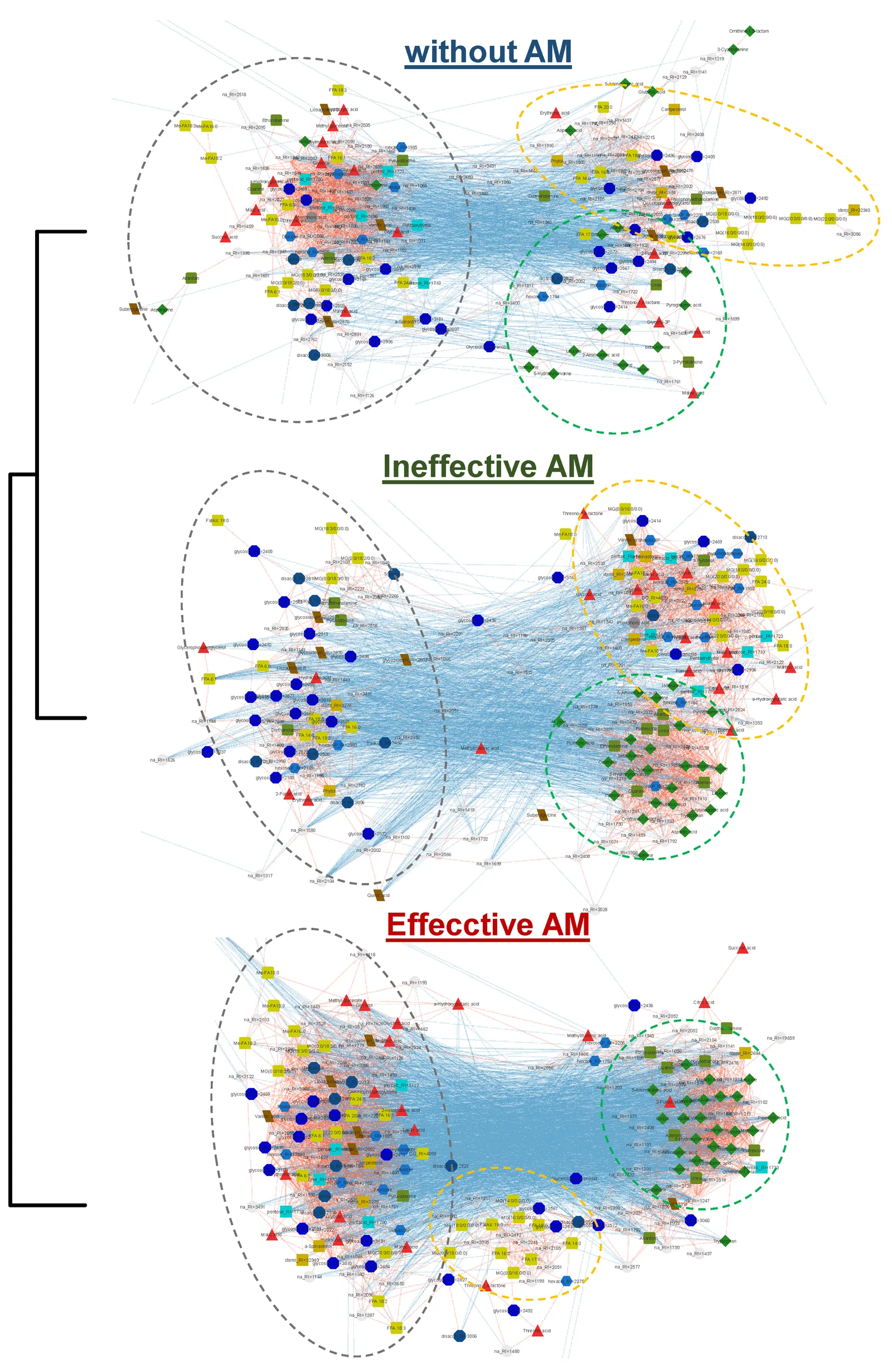
Mapping of metabolites based on strong correlations (*r* > 0.7, FDR < 0.05). The dendrogram on the left shows clustering of networks by edge sets. Gray dashed lines—metabolites from different classes; green dashed lines—amino acids; orange dashed lines—FFAs and acylglycerols.

To assess the effect of mycorrhization with AM fungi of different efficiency on the functional interrelationships of metabolite pools, we performed mapping of metabolites based on strong correlations of their levels. Figure 7 presents graphs in which nodes correspond to metabolites and edges to strong correlations (*r* > 0.7, FDR < 0.05). The graph was constructed such that positive connections attract metabolites proportionally to the correlation value. The constructed networks differed both in appearance and in characteristics (Table 1). It is noticeable that mycorrhization is accompanied by an increase in the number of strong correlations. This effect is much more pronounced in the case of effective symbiosis. As the number of connections increases, the density increases, while the network diameter, network radius, and characteristic path length decrease. Effective symbiosis is also associated with an increase in the clustering coefficient and network centralization. Network heterogeneity was highest in plants without AM. Additionally, in all networks, positive correlations outnumbered negative ones. Notably, the ratio of positive to negative correlations decreased in the effective mycorrhization treatment, whereas ineffective AM did not produce such an effect.

The networks were heterogeneous. A large region consisting of metabolites from different classes could be distinguished (gray dashed line at the top). The structure of the second group (at the bottom of the graph) depended on mycorrhization status. In the non-AM treatment, it consisted of two closely adjacent regions: the first containing numerous amino acids (green dashed line at the bottom) and the second containing FFAs and acylglycerols (orange dashed line at the bottom). In the ineffective mycorrhization treatment, these regions became more distinct, and in the effective mycorrhization treatment, this trend was amplified, with the amino acid and fatty acid clusters diverging.

## 3. Discussion

### 3.1. Contrasting Phenotypes of Medicago lupulina Under Inoculation with AM Fungi of Different Efficiency

In the present study, a comparative analysis of AM development and root metabolic rearrangements is provided for the first time in the highly responsive *Medicago lupulina* line MlS-1 inoculated with two strains contrasting in symbiotic efficiency: the effective *Rhizophagus irregularis* strain RCAM00320 and the ineffective *Glomus* sp. strain 129.1Te. The obtained results demonstrate two distinct symbiotic interactions, which is consistent with current concepts of the mutualism—parasitism continuum in AM symbiosis, resulting from fine-tuned regulation of plant metabolic pathways [27]. According to this concept, AM symbiosis varies widely from mutualistic to antagonistic interactions between myco- and phytosymbionts, depending on the genotype combination of partners as well as environmental conditions (phosphorus form and availability). Nevertheless, substantial differences should be noted between the PMS models with ineffective AM discussed here: “*Medicago lupulina* + *Glomus* sp.” (the present study) and “*Sorghum caudatum*/*S. bicolor* + *Gigaspora gigantea*” [27]. In the first PMS, symbiotic efficiency was zero (no effect of AM on host plant growth performance; MGR calculated by fresh weight per plant ranged from −2% to +3% at 21–35 DAS), whereas in the second PMS, MGR calculated by shoot dry weight was significantly negative, ranging from −42% to −70% at 30 DAS. That is, *Gigaspora gigantea* isolate MN922A exerted a significant negative (pathogenic) effect on the growth of both *Sorghum caudatum* accession PI-297130 and *S. bicolor* line PI-562730.

Meanwhile, studies on the role of AM fungi in adaptation to biotic stress factors have mostly shown that mycorrhization generally promotes protection against pathogenic fungi (*Alternaria*, *Fusarium*, *Phytophthora*, *Pythium*, *Rhizoctonia*, and *Verticillium*), pathogenic bacteria (*Ralstonia solanacearum* and *Pseudomonas syringae*), and even nematodes (*Pratylenchus* and *Meloidogyne*) and insects (*Otiorhynchus sulcatus*). It is known that AM reduces infection by fungal pathogens by 30–42% and by nematodes by 44–57% [4]. The protective effect is based on the activation of plant secondary metabolism, including the accumulation of defensive compounds such as alkaloids and terpenoids, as well as the initiation of an immune response in the host plant after recognition of pathogenic fungi upon detection of their effectors (signaling molecules), after which AM fungi produce lipo-chitooligosaccharides and short chitooligosaccharides that are transmitted from cell to cell through plasmodesmata and activate systemic acquired resistance and/or induced systemic resistance [4]. On the other hand, under antagonistic interactions in ineffective AM symbiosis, similar effects are observed, for example, in the PMS “*S. caudatum* accession PI-297130 & *S. bicolor* accession PI-562730 + *G. gigantean* isolate MN922A”, namely, accumulation of the antifungal compound *p*-hydroxyphenylacetaldoxime, an intermediate of dhurrin biosynthesis with fungicidal properties [27].

Plants inoculated with the effective strain RCAM00320 exhibited a high MGR, calculated by fresh plant biomass and reaching maximum values (about 200%) at the branching initiation (35 DAS) and flowering initiation (48 DAS) stages (Figure S1A). This confirms the formation of a highly effective plant-microbe system, as previously described for this model line [24]. In contrast, plants inoculated with the ineffective strain 129.1Te did not show significant symbiotic efficiency. It can be hypothesized that the lack of growth response is associated with slower development of symbiotic structures (delayed growth of AM fungal symbiotic structures) in the 129.1Te inoculation treatment (Figure S1C). Thus, MGR values were close to zero during the vegetative stages of host plant development and reached only +16.2% by the fruiting initiation stage (54 DAS). At the same time, during the vegetative stages (21 and 35 DAS), differences in the RFW: SFW ratio (Figure S1B) between the control and both mycorrhizal treatments were significant. This may indicate adaptive responses of non-AM plants, which form a more extensive root system under phosphorus deficiency compared to shoot development [29,30,31]. Ineffective AM symbiosis did not compensate for the deficiency in root system development, nor for the Pi deficiency in the substrate, which was reflected in its low MGR (Figure S1A).

Differences in arbuscule abundance (*A*, %), the main symbiotic structures ensuring nutrient exchange between partners [29], were statistically significant at all studied stages (*p* < 0.05; Figure S1C). Effective symbiosis was characterized by high and rapidly increasing arbuscule abundance during 21–35 DAS, reaching 34% by the fruiting initiation stage (54 DAS), whereas ineffective symbiosis showed a pronounced delay with very low values at the vegetative stages (less than 1%) followed by an increase at reproductive stages (up to 18%) (Figure S1C). These results indicate delayed or impaired development of functionally important symbiotic structures upon inoculation with the ineffective strain, which may be related to the specific functioning of fungal effector proteins (such as the SP7 protein in *R. irregularis* identified in PMS with *Medicago truncatula*, *Nicotiana benthamiana*, and *Allium schoenoprasum*) or alternatively to the fungus’s inability to effectively suppress host plant defense responses [32,33,34]. The contrasting phenotypes observed in the two PMS served as the basis for subsequent metabolomic analysis, which allowed the identification of specific metabolic markers associated with symbiosis efficiency (see Section 3.5).

### 3.2. Effective Mycorrhization as a Driver of Root Metabolome Rearrangement

AM formation, regardless of its efficiency, significantly altered the root metabolomic profile of *Medicago lupulina* at all stages of host plant development; that is, mycorrhization led to pronounced ontogenetic changes in the metabolome (Figure 2 and Figure S2). PCA (Figure 1 and Figure 2) showed that mycorrhization with the effective strain RCAM00320 led to the most substantial metabolic rearrangements. Thus, effective AM symbiosis induced more pronounced ontogenetic changes in the metabolome (44% variance explained on PC1 vs. 35% in the control and 36% under ineffective AM; Figure 1), indicating profound metabolic reprogramming. Overall, the obtained data are consistent with the assessment of the effect of inoculation with strain RCAM00320 on the leaf metabolome of *M. lupulina*, where it was shown that mycorrhization with the effective strain was the determining factor in metabolic rearrangements [24]. Meanwhile, the present study showed that phase-related changes in the root metabolome in the “without_AM” and “Ineff_AM” treatments were largely similar (Figure 1) but less pronounced than in the “Eff_AM” treatment.

At the early stage (21 DAS), effective mycorrhization exerted a more pronounced effect on the plant metabolome, explaining 58% of metabolomic data variance vs. 44% for ineffective mycorrhization, indicating a faster and deeper metabolic rearrangement. Over time, these differences diminished, and by 48 and 54 DAS, the metabolic impact of both symbioses became comparable, suggesting that the ineffective fungus eventually triggers some common metabolic pathways but with a significant delay.

No similar studies assessing metabolomic dynamics at multiple time points for effective and ineffective AM symbiosis have been conducted. However, root metabolome assessment at 30 DAS was carried out in effective model PMS “*Sorghum caudatum* + *Rhizophagus irregularis*” and “*S. bicolor* + *R. irregularis*” compared with ineffective PMS “*S. caudatum* + *Gigaspora gigantea*” and “*S. bicolor* + *G. gigantea*” [27]. The study by Kaur et al. also showed similarities in some metabolic changes between effective and ineffective symbioses in sorghum, which is consistent with our data at vegetative stages in the effective PMS “*M. lupulina* + *R. irregularis*” compared with the ineffective PMS “*M. lupulina* + *Glomus* sp.”. Another study of PMS “*Solanum lycopersicum* + *Rhizophagus irregularis* & *Funneliformis mosseae*” [35] at 19, 23, 27, and 35 DAS shows that the system under study is ineffective, with the negative effect of the AM fungus being maximal at the first time point.

To explore the relationship between the metabolome and mean arbuscule abundance, we constructed an OPLS model and performed regression using the Random Forest method (Figure S3). The OPLS model parameters were Q^2^Y = 0.88, *p* ≤ 0.01. The predictive component accounted for 15% of the variance. Figure S3 shows a bar plot of the predictive component loadings, where positive loadings correspond to an increase in metabolite levels with increasing arbuscule abundance. Unsurprisingly, increased arbuscule abundance was accompanied by higher phosphate levels. In addition, increased arbuscule abundance was associated with lower FFA levels and higher accumulation of acylglycerols. Levels of glycerol and its phosphorylated forms also increased, as did trehalose, sucrose, fructose, and glucose. Collectively, these trends may indicate enhanced carbon transfer to the fungus in exchange for phosphate. Some glycosides, putatively containing aromatic groups, showed a tendency to increase. Such molecules may play a role in plant-fungus communication. An increase in sterol levels was also observed, indirectly suggesting increased membrane activity.

### 3.3. Reprogramming of Central Carbon and Energy Metabolism

Our OPLS-DA and MSEA analysis revealed a key metabolic feature of effective AM symbiosis: the sequential suppression of the pool of TCA cycle intermediates, such as citrate, succinate, and malate, especially at the early stage (21 DAS) (Figure 3). This suppression of the TCA cycle has not previously been characterized in detail in the context of comparing effective and ineffective symbiosis. Previous studies have shown a multidirectional effect of mycorrhization on TCA cycle content, an increase in monocots and a decrease in some intermediates in dicots, but a clear link with AM symbiosis efficiency, as well as with host plant developmental stage, has not been demonstrated [36]. TCA cycle suppression has been described for roots in the effective PMS “*M. lupulina* + *R. irregularis*” under various phosphorus nutrition conditions [11], as well as for some other dicots, for example, in the PMS “*Lotus japonicus* + *Funneliformis mosseae*”, but in leaves [37]. It is believed that this suppression results from inhibition of early stages of the Krebs cycle in combination with intensification of transamination reactions, leading to rapid depletion of the ketoacid pool and redirection of organic carbon toward amino acid synthesis [38,39]. Amino acid accumulation is observed, for example, in the effective PMS “*Wedelia trilobata* + *Glomus versiforme*” at ~60 DAS (MGR calculated by shoot dry biomass = 51%; [40]). Confirmation of the shift toward glycolysis stimulation and Krebs cycle suppression upon mycorrhization comes from the study by Thokchom et al. [41] in the PMS “*Ocimum tenuiflorum* + *R. irregularis*”, in which inoculation with AM fungus is shown to stimulate photosynthesis and promote redirection of carbon resources from the Krebs cycle toward secondary metabolism, ultimately leading to higher accumulation of essential oils.

In parallel with TCA cycle suppression, we observed activation of amino acid metabolism at 21 DAS, including accumulation of leucine, glycine, lysine, and other amino acids (Figure 3). Similar AM-induced increases in certain amino acid pools (largely lysine) are recorded in roots of *M. truncatula* [39], as well as glutamic acid, histidine, and cysteine in *Solanum lycopersicum* [20], and largely aspartic and glutamic acids in *Anchusa officinalis* [42]. This accumulation may serve multiple functions: it may provide a rich nitrogen source for the fungus, supply resources for fungal effector protein synthesis, or represent a nitrogen reserve localized in the root and resulting from improved overall nitrogen assimilation.

In the study by Kaur et al. [27], analysis of the parasitic phenotype of AM symbiosis in sorghum (*Sorghum caudatum* and *S. bicolor*) reveals accumulation of compounds suppressing fungal partner development (such as *p*-hydroxyphenylacetaldoxime and acetyl glycoside of kaempferol–luteolin). In our work, ineffective symbiosis was characterized not only by lower arbuscule abundance (Figure S1C) but also by distinct individual metabolic patterns: in the effective AM treatment compared with ineffective AM, reduced accumulation of FFAs 16:1 was observed only at the early stage (21 DAS), whereas at 35, 48, and 54 DAS, effective symbiosis was associated with greater accumulation of both FFAs 16:1 and monoacylglycerols, particularly at the branching initiation stage (35 DAS). This difference in lipid metabolism patterns appears to reflect different intensities of metabolic exchange between partners and will be discussed in detail in the context of marker compounds in Section 3.5.

Comparative analysis of metabolic profiles of effective and ineffective AM symbioses in *M. lupulina* revealed the dynamic nature of γ-aminobutyric acid (GABA) accumulation. At the early stage of symbiosis development (21 DAS), the effective PMS “*M. lupulina* + *R. irregularis*” exhibited higher GABA content, whereas at later time points (35, 48, and 54 DAS), its level was higher in the ineffective variant “*M. lupulina* + *Glomus* sp.”. These results are consistent with data from Kaur et al. [27], showing negative regulation of the GABA pool and proteinogenic amino acids in the highly effective PMS “*S. caudatum* + *R. irregularis*” at 30 DAS. Under conditions of weak response to inoculation in *Triticum durum*, a decrease in the total amino acid pool is observed to be coupled with a metabolic shift toward GABA synthesis [43]. Under salt stress in *Zea mays*, inoculation with *F. mosseae* is accompanied by increased accumulation of organic acids, including some TCA cycle intermediates [44]. According to current concepts, salt stress can inhibit the activity of key TCA cycle enzymes. However, activation of the GABA shunt can compensate for this inhibition, providing an alternative pathway for carbon entry into mitochondria, bypassing salt-sensitive enzymes and supporting enhanced respiration [45]. Consequently, the low content of GABA and TCA cycle intermediates in the effective PMS “*M. lupulina* + *R. irregularis*” may reflect adaptation to stress, involving regulatory changes in carbon and nitrogen metabolism under phosphorus deficiency.

Thus, reprogramming of central carbon and energy metabolism, including TCA cycle suppression, modulation of amino acids and GABA pools, and activation of lipid metabolism, creates the metabolic background on which specific marker patterns of effective symbiosis are formed (see Section 3.5). However, the key differences between effective and ineffective AM are most clearly manifested at a specific developmental stage.

### 3.4. Branching Initiation Stage as a Critical Metabolic Transition

The results of metabolomic analysis indicated that the branching initiation stage (35 DAS) is supposed to be a key stage for metabolic divergence between effective and ineffective AM symbioses. At this stage, effective AM symbiosis triggered the most significant enhancement of glycerolipid, fatty acid, and sterol accumulation, accompanied by suppression of the TCA cycle and amino acid metabolism (Figure 4), which coincided with maximal MGR (Figure S1A). Importantly, in the ineffective AM treatment at the same stage, arbuscule abundance was minimal (Figure S1C), indicating a metabolic “bottleneck” preventing the transition of the fungus to a symbiotically beneficial interaction with the host plant. Ineffective AM was characterized, conversely, by accumulation of TCA cycle intermediates and amino acids.

Notably, starting from the branching initiation stage, the content of 16-carbon free fatty acids (FFAs 16:1) was significantly higher in the effective PMS “*M. lupulina* + *R. irregularis*” compared with the ineffective PMS “*M. lupulina* + *Glomus* sp.”. The content of FFAs 16:0 showed significantly less pronounced dynamics. Meanwhile, it is known that FFAs 16:0 and 16:1 are synthesized exclusively by the intraradical mycelium of AM fungi in mycorrhizal roots [46], indicating that FFAs 16:0 and 16:1 may play important roles in the obligate biotrophy of AM fungi. FFA 16:1ω5 is the main biomarker of AM fungal biomass [47]. Thus, the higher level of FFA 16:1 in the effective PMS at 35, 48, and 54 DAS characterizes close metabolic exchange between symbiosis partners. These compounds, along with other lipid metabolites, will be considered as potential markers of AM efficiency in Section 3.5.

Previously, Xie et al. [5] showed that in *M. truncatula*, expression of key symbiotic markers (fungal phosphate transporter RiPT7 and plant transporter MtPT4) is high at 42 DAS, which approximately corresponds to the studied branching initiation stage. Our metabolomic data provided functional physiological confirmation of these transcriptomic observations. Furthermore, in a recent study by Yurkov et al. [11], it is also shown that the branching initiation stage is crucial for the manifestation of mycorrhization and phosphorus nutrition effects on the root metabolome in the effective PMS “*M. lupulina* + *R. irregularis*”, under both phosphorus deficiency and optimal phosphorus nutrition, which is fully consistent with the conclusions of the present work.

Thus, the branching initiation stage is characterized by a critical metabolic transition determining the subsequent fate of symbiotic relationships. This metabolic bottleneck hypothesis is supported by He et al. [48], who showed that Myc-factor-induced root development in rice depends on LysM-receptor kinases OsCERK1/OsMYR1. Impaired signal perception, as observed in receptor mutants [48], likely limits downstream metabolic reprogramming, consistent with our metabolomic data, where ineffective symbiosis exhibited TCA cycle accumulation and perturbed amino acid regulation. At the branching initiation stage, the main differences in metabolic profiles are established, which can subsequently be used to identify specific markers of effective AM symbiosis.

### 3.5. Metabolite Markers of AM Symbiosis Efficiency

Detailed comparison of DAMs at effective and ineffective AM variants allowed the identification of a number of compounds that might serve as specific markers of symbiotic efficiency in the model PMS “*M. lupulina* + *R. irregularis*”. The proposed marker set covers various compound classes and demonstrates stable accumulation patterns depending on host plant developmental stage.

**Carbohydrate markers:** at all four studied developmental stages (21, 35, 48, and 54 DAS), roots of plants with effective AM showed significantly higher trehalose accumulation compared with the ineffective AM treatment (Figure 3, Figure 4, Figure 5 and Figure 6). This result is consistent with the known role of trehalose as one of the main transported carbohydrates in AM symbiosis, synthesized by the fungal partner from plant glucose [49,50]. The consistently high level of this disaccharide in the effective PMS indicates intensive and stable carbon exchange between partners, a distinctive feature of mutualistic interaction [51]. Conversely, the low trehalose content in the ineffective treatment at all stages reflects limited carbon flow to the fungus, correlating with low arbuscule abundance (Figure S1C) and absence of growth response.

Furthermore, hexose-P_RI = 2383, hexose-P_RI = 2206, and pentalc_RI = 1719 were consistently higher in effective AM at three of the four developmental stages, pointing to a broader reprogramming of carbohydrate and phosphorus metabolism. The elevated pools of phosphorylated hexoses and a pentose alcohol suggest enhanced carbohydrate turnover or altered carbon allocation in effectively mycorrhizal roots, potentially supporting both fungal nutrition and plant adaptive responses under Pi deficiency.

**Glycosides as potential markers:** are of particular interest due to the divergent dynamics of glycoside accumulation (Figure 3, Figure 4, Figure 5 and Figure 6), which allows these compounds to be considered as potential markers of effective AM symbiosis. However, complete identification of these metabolites and their role in AM development requires further investigation. Glycoside_RI = 2436 was stably accumulated in effective AM at three of the four analyzed developmental stages, highlighting the rearrangement of carbohydrate and phosphorus metabolism. At the early stage (21 DAS), the content of glycoside_RI = 3095 in effective AM was lower than in ineffective AM; however, starting from the branching initiation stage (35 DAS), significantly higher accumulation of this metabolite was observed in effective symbiosis. This dependence for glycoside_RI = 3095 was revealed for the first time, allowing it to be considered as a novel potential indicator of effective AM symbiosis, consistent with the key role of the branching stage as a metabolic “switch” (see Section 3.4). In contrast, the content of glycoside_RI = 3169 in roots with effective AM was consistently lower than in the ineffective treatment at all four time points (Figure 3, Figure 4, Figure 5 and Figure 6). Its elevated accumulation in ineffective symbiosis may reflect the synthesis of secondary metabolites of a defensive nature, consistent with data from Kaur et al. for ineffective PMS “*S. caudatum* + *G. gigantea*” and “*S. bicolor* + *G. gigantea*” [27].

It is known that glycosides play important roles in AM symbiosis. Glycosylated blumenols are accumulated in the roots of most mycorrhizal plants and serve as specific markers of colonization; their accumulation correlates with the degree of symbiosis development and is not induced by other stressors [52]. In tomato, it has been shown that the AM-specific UDP-glucosyltransferase SlUGT132, which glycosylates flavonoids, negatively regulates AM development, indicating the involvement of glycosides (astragalin, tiliroside, and cynaroside) in symbiosis autoregulation [53]. Thus, the glycoside accumulation patterns revealed in our study are consistent with their known role in AM symbiosis regulation; however, definitive conclusions require their identification.

**Lipid and sterol markers:** the following compounds emerged as stable positive markers of effective symbiosis at three or all four time points (Figure 3, Figure 4, Figure 5 and Figure 6):

− Campesterol and sterol_RI = 3279—their elevated accumulation in effective AM indicates sterol accumulation in host plant roots, which may be attributed to arbuscule formation and membrane remodeling [54];
− Glycerol-3P, a key lipid metabolism metabolite, is consistent with the overall enhancement of glycerolipid metabolism under effective mycorrhization;
− Glycerophosphoglycerol showed consistently higher levels in effective AM at three of the four developmental stages (except at 21 DAS), highlighting the rearrangement of membrane lipid metabolism.

**Markers with changing dynamics:** of interest are metabolites for which lower accumulation was observed in effective AM compared with ineffective AM at the early vegetative stage, but starting from 35 DAS, the relationship reversed. These compounds include citric acid, hexacid_RI = 2375, guanine, and glutamine (Figure 3, Figure 4, Figure 5 and Figure 6). For glutamine and guanine, this dynamic may indicate early mobilization of nitrogenous compounds by the fungus, followed by normalization or even stimulation of their pool upon establishment of effective metabolic balance in the PMS.

**Vegetative stage markers:** the most pronounced differences between effective and ineffective symbiosis in favor of effective AM were observed at the vegetative stages (21 and 35 DAS) and gradually diminished toward the reproductive stages (48–54 DAS), which is particularly evident for the group of metabolites including sterol_RI = 2684, Me-FA18:3, MG(0:0/16:0/0:0), MG(14:0/0:0/0:0), and MG(16:0/0:0/0:0) (Figure 3, Figure 4, Figure 5 and Figure 6). This trend correlated with faster arbuscule development in the effective treatment at vegetative stages (Figure S1C) and subsequent delayed increase in arbuscule abundance in the ineffective treatment during reproductive stages. Thus, metabolomic profiles of vegetative stages were the most informative to investigate AM symbiosis efficiency, consistent with the conclusions on the critical role of the branching initiation stage (see Section 3.4) and previously published data for the effective PMS “*M. lupulina* + *R. irregularis*” [11].

The obtained results are in good agreement with studies conducted on other model systems. In sorghum, Kaur et al. [27] also showed that effective AM symbiosis with *R. irregularis* differs from ineffective AM with *G. gigantea* in fatty acid and carbohydrate content; however, that study failed to identify unambiguous metabolic markers common to the two sorghum lines. Our study on *M. lupulina* using contrasting strains of the same species (effective *R. irregularis* strain RCAM00320 and ineffective *Glomus* sp. strain 129.1Te) allowed the identification of a more stable set of marker metabolites, emphasizing the importance of proper model PMS selection. A number of metabolites identified in our study as markers (campesterol, glycerol-3P, phosphorylated hexoses) are consistent with data from Rivero et al. [20] on tomato, where mycorrhization with different AM strains caused contrasting rearrangements of lipid and carbohydrate metabolism. Furthermore, our results complement recent metabolomic studies on *M. truncatula* [39] and *Pisum sativum* [23], showing that specific efficiency markers may be species-specific and depend on developmental stage, but some general patterns (e.g., the role of trehalose and fatty acids) are conserved. In view of these data obtained across different plant-microbe systems, it is reasonable to assume that the observed changes in the levels of marker metabolites are likely the result of active metabolic reprogramming by the plant, required to support effective AM.

Thus, the proposed set of metabolites may serve as a basis for developing metabolomic markers of symbiotic efficiency, which could potentially be used for screening AM fungal strains and assessing their agronomic potential under field conditions.

The identified marker metabolites not only characterize AM symbiosis efficiency at the level of individual compounds but also reflect the profound reorganization of metabolic networks that occurs during effective AM formation. Analysis of correlative relationships between metabolites allows this reorganization to be assessed at the systemic level and to identify structural changes in metabolic networks caused by mycorrhization.

### 3.6. Reorganization of Metabolic Networks Under Mycorrhization

The identification of individual marker metabolites was complemented by analysis of metabolic networks through mapping of metabolites based on strong correlations of their levels. This approach is commonly used for visualization of metabolic connections [55,56,57]. It is known that correlation patterns exhibit specificity according to environmental conditions [58,59,60,61] and developmental stage [62]. It has previously been shown that effective AM affects the correlation of metabolite networks in leaves [24] and roots [11] of black medic. In the present case, we mapped metabolites based on Spearman correlations of their normalized content in samples. As shown in Figure 7, ineffective AM exerted a relatively weak effect on the correlation pattern. Moreover, the network constructed for plants with ineffective AM was closer to that for non-AM plants.

The most interesting observation was the divergence of amino acid (green) and fatty acid (yellow) clusters under effective mycorrhization, whereas in the control these clusters were closely linked (Figure 7). This functional specialization may reflect symbiosis-controlled transfer of these compounds to the fungal partner. On the one hand, the separation of the amino acid cluster may be driven by enhanced protein synthesis associated with faster growth of effectively mycorrhizal plants. On the other hand, the segregation of the fatty acid cluster may be related to carbon supply to the fungus in the form of acylglycerols. It is known that AM fungi are lipid auxotrophs and depend on their host plants for fatty acid supply [46,63,64]. This is consistent with the fact that effective AM symbiosis is accompanied by significant positive regulation of glycerolipid metabolism and sterol accumulation, corresponding to the need for enhanced lipid synthesis to support fungal growth and arbuscule development [54].

Under effective AM, the number of strong correlations increased almost twofold, whereas under ineffective AM it increased by only one-quarter (Table 1). Concurrently, associated characteristics such as average number of neighbors and network density increased. At the same time, network diameter, network radius, and characteristic path length decreased, with effects being more pronounced in the case of effective AM. In control plants experiencing phosphorus deficiency, the predominance of positive correlations between metabolites likely reflects a coordinated stress-induced response of the metabolic network—passive covariation of metabolites that is a direct consequence of phosphorus deficiency [65]. Under varying phosphorus availability [11], the decrease in the ratio of positive to negative correlations under effective AM indicates a transition from passive stress response to active regulatory control mediated by the symbiont. Since correlations result from the combination of all reactions and regulatory processes [66,67,68], the increase in their number likely occurs due to the emergence of a new regulatory circuit during effective AM formation.

High positive correlation may reflect proximity of metabolites to equilibrium and a high level of metabolic flux [66,69], whereas negative correlations are determined by the law of energy conservation [66]. The decrease in the ratio of positive to negative correlations under effective AM may result from intensive efflux of substances to the fungal partner.

The results of our study have identified key directions for future research aimed at expanding the range of symbiotic systems with varying efficiency.

## 4. Materials and Methods

### 4.1. Biological Materials, Inoculum Preparation, and Cultivation Conditions

The *Medicago lupulina* L. subsp. *vulgaris* line MlS-1, which is characterized by high response to AM fungal inoculation under phosphorus-deficient conditions, was used in this study. Two AM fungal strains differing in efficiency were used for inoculation: the highly effective *Rhizophagus irregularis* strain RCAM00320 (Błaszk., Wubet, Renker & Buscot) C. Walker & A. Schüßler and the ineffective *Glomus* sp. strain 129.1Te. Both strains were obtained from the Laboratory of Ecology of Symbiotic and Associative Rhizobacteria at the All-Russia Research Institute for Agricultural Microbiology (ARRIAM), and their MGR was previously characterized [28,70], with the species identity of *R. irregularis* confirmed by molecular genetic methods [71]. Since AM fungi are obligate biotrophs, stock cultures of both strains were maintained on roots of *Plectranthus verticillatus* (L.f.) Druce under conditions preventing cross-contamination.

Inoculum preparation involved four consecutive steps according to a previously developed protocol [24]. First, *Plectranthus* cuttings were surface-sterilized with 0.5% sodium hypochlorite (60 min) followed by three rinses with sterile distilled water (30 min each). Sterile cuttings were then inoculated with two root fragments (10 mm each) from donor mycorrhizal plants (stock culture) and planted in a low-Pi substrate. After 180 days of cultivation under conditions identical to the main experiment, the root mass was harvested, cut into 10 mm fragments, and thoroughly mixed to obtain a homogeneous inoculum.

The experiment was conducted under sterile conditions using a microvegetation method to prevent spontaneous infection by rhizobia and other microsymbionts. The growth chamber was pre-irradiated with ultraviolet light; the soil-sand substrate (2:1 *v*/*v*) and all tools were autoclaved at 134 °C and 2 atm (1 h, repeated after 2 days to eliminate potential toxicity). Seeds of *M. lupulina* were scarified with concentrated sulfuric acid (5 min), then stratified at +5 °C (1 day) and germinated at +27 °C in the dark (2 days). Selected equal-sized seedlings were planted two per vessel containing 210 g of substrate. Inoculation of *M. lupulina* seedlings was performed at planting using inoculum containing an average of 100 AM fungal vesicles per black medic plant (2 fragments of 10 mm each). The agrochemical characteristics of the soil were as follows: sod-podzolic loamy soil with low available phosphorus (23 mg P_2_O_5_/kg according to Kirsanov), potassium (78 mg K_2_O/kg), organic matter 3.64%, pH_KCl_ 6.4, pH_H2O_ 7.3. Plants were watered with boiled water every other day to 60% of full water-holding capacity [11]. The light regime was 16 h/8 h at 25–27 °C. The treatment scheme included three variants: (1) inoculation with the effective strain RCAM00320 (“Eff_AM”); (2) inoculation with the ineffective strain 129.1Te (“Ineff_AM”); (3) application of sterilized non-mycorrhizal *Plectranthus* roots (“Without_AM”, control). Inoculation coincided with seedling planting. Sampling for biochemical and microscopic analyses was performed at four developmental stages: 21 days (2nd true leaf), 35 days (branching initiation), 48 days (flowering initiation), and 54 days (fruiting initiation) after sowing and inoculation (DAS). In all inoculation treatments, the developmental stages of the analyzed plants coincided. For each biological replicate (n = 3 per treatment), the root system from eight plants was collected, fresh root and shoot weights were determined, and roots were immediately frozen in liquid nitrogen and stored at −80 °C until analysis.

### 4.2. Assessment of Arbuscule Abundance in Roots and Calculation of Mycorrhizal Growth Response (MGR)

Microscopic assessment of colonization was conducted according to the method of Phillips and Hayman [72] with modifications. Root samples were air-dried, macerated, and stained with 0.05% trypan blue in a mixture containing lactic acid (90%), glycerol, and distilled water in a volumetric ratio of 62:63:875, respectively, with 0.3 g of dye per liter. Preparations were made as crushed root fragments of 1 cm length and analyzed using a Stemi 2000 stereomicroscope (Zeiss, Oberkochen, Germany) and an Olympus CX43 microscope (Olympus, Hachioji, Tokyo, Japan) at 10× magnification (Plan Achromat PLCN10X-1-7 objective, WD 10.5 mm, Olympus, Hachioji, Tokyo, Japan). For each treatment, at least 8 biological replicates were processed. Arbuscule abundance in roots (*A*, %) was determined according to the standard method [73] using the computer program developed by Vorobyev et al. [74].

The mycorrhizal growth response (MGR, AM efficiency) was assessed by the increase in fresh plant biomass relative to the non-AM control. MGR was calculated using the formula:

MGR = ((AM+) − (AM−))/(AM−) × 100%

where “AM+” is the biomass of mycorrhizal plants and “AM−“ is the biomass of non-AM plants.

### 4.3. Gas Chromatography—Mass Spectrometry (GC-MS)

For metabolite profiling, root samples (100 mg) were collected at each of the four developmental stages and immediately frozen in liquid nitrogen. Material homogenization was performed using a MM 400 ball mill (Retsch GmbH, Haan, Germany). Metabolite extraction was carried out in methanol in a volume of 2 mL with incubation on a TS-100C thermoshaker (BioSan SIA, Riga, Latvia) at 10 °C and 900 rpm for 30 min. After centrifugation (12,000× *g*, 10 min, 4 °C), the supernatant was separated and evaporated in a CentriVap vacuum concentrator (Labconco Corporation, Kansas City, MO, USA). The dry residue was dissolved in pyridine; BSTFA:TMCS (99:1, Merck KGaA (Sigma-Aldrich), Darmstadt, Germany) was added, and derivatization was conducted at 90 °C for 20 min. Tricosane (nC_23_; 0.25 mg/mL) was used as an internal standard.

Analysis was carried out on an Agilent 6850 chromatograph with an Agilent 5975 quadrupole detector (Agilent Technologies, Santa Clara, CA, USA) controlled by MassHunter software v. 10.1 (Agilent Technologies, Santa Clara, CA, USA) using a DB-5ms capillary column (Agilent Technologies, Santa Clara, CA, USA) at a helium flow rate of 1 mL/min. Chromatographic conditions: the inlet was operated in splitless mode at 250 °C; the temperature gradient was from 70 °C to 320 °C at a rate of 6 °C/min. Electron impact ionization energy was 70 eV, and the ion source temperature was 230 °C. Data were processed using PARADISe software v. 6.0.1 (UCPH FOOD, Copenhagen, Denmark) [75] in conjunction with the NIST MS Search mass spectral library system (Gaithersburg, MD, USA) [76]. Additionally, metabolite deconvolution and identification were carried out using the AMDIS system (Automated Mass Spectral Deconvolution and Identification System, Gaithersburg, MD, USA). Compound identification was based on comparison of the obtained mass spectra and Kovats retention indices with data from the NIST2020 and Golm Metabolome Database (GMD) libraries [76], as well as using an in-house database created in the laboratory of analytical phytochemistry with financial support of BIN RAS (#124020100140-7).

### 4.4. Statistical Analysis

Statistical significance of differences for fresh biomass, MGR, and mycorrhization (arbuscule abundance) was assessed using One-Way Analysis of Variance (ANOVA) and Tukey’s HSD test at a significance level of *p* < 0.05. Metabolomic data were normalized by the median of each observation and log-transformed. For multivariate analysis, the data were autoscaled. Outlier detection and removal were performed using Dixon’s test (*outliers* package v. 0.15) [77]. Missing values were imputed using the k-nearest neighbor (KNN) method with the *impute* package v. 1.74.1 [78]. For visualization of multivariate relationships, Principal Component Analysis (*pcaMethods* v. 1.92.0) [79] and multidimensional scaling (MDS) [80] were applied. The significance of groupings from MDS and PCA was confirmed by a PERMANOVA test (*adonis2* function from *vegan* v. 1.6–10; 10^5^ permutations, Euclidean distance) [81]. Discriminant analysis was carried out using Orthogonal Partial Least Squares Discriminant Analysis (OPLS-DA, *ropls* package v. 1.32.0). Models were validated by Q^2^ (*p* ≤ 0.03). Models’ parameters were pooled in Supplementary Materials (Table S3). Variables contributing most to group separation were identified by VIP (Variable Importance in Projection) values according to Thévenot et al. [82]. Metabolite set enrichment analysis was conducted using the *fgsea* algorithm v. 1.26.0 [83] based on KEGG (Kyoto Encyclopedia of Genes and Genomes) annotations [84], retrieved via the *KEGGREST* package v. 1.40.1 [85] using *M. truncatula* as the reference genome. Metabolite lists for pathways were manually adjusted by excluding poorly represented or overly large sets, and compounds identified at the class level (hexoses, disaccharides, etc.) were assigned to the corresponding generalized pathways. Correlation networks were constructed in Cytoscape v. 3.10.3 [86].

## 5. Conclusions

To summarize, this comparative analysis of a plant-microbe system forming effective and ineffective AM symbiosis under Pi deficiency across two vegetative and two reproductive stages revealed several key features of AM development in *M. lupulina*. Effective symbiosis was characterized by adaptation to Pi deficiency, a pronounced high MGR (up to 200%), and significant alterations in the root metabolic profile, whereas ineffective symbiosis did not confer a significant MGR. A key feature of effective AM was found to be substantial changes in plant metabolism at the critical early branching stage. This stage defined the metabolic transition that dictated the subsequent development of the symbiosis. The metabolic traits of effective AM included negative regulation of the TCA cycle (shortage of the intermediate pool of citrate, malate, and others), accumulation of trehalose, phosphoric acid, and a range of phosphorylated metabolites, activation of lipid metabolism, and increased levels of FFAs 16:1 and other fatty acids, particularly during the reproductive stages. In contrast, ineffective AM symbiosis was characterized by delayed metabolic rearrangement with MGR close to zero, including accumulation of TCA cycle intermediates, and disruption of amino acid metabolism regulation.

An important outcome was the identification of novel metabolic markers of effective symbiosis, including specific patterns of fatty acid accumulation in the effective AM compared with the ineffective AM, as well as a rearrangement of correlative relationships between metabolite pools, expressed as a divergence of amino acid and fatty acid clusters. These changes may reflect redistribution of metabolites between symbiosis partners in favor of the mycobiont, but may also result from adaptive rearrangements of plant metabolism aimed at maintaining symbiotic relationships.

## Figures and Tables

**Table 1:** Correlation network characteristics.

Correlation Networks Characteristic	Without_AM	Ineff_AM	Eff_AM
Number of nodes	225	228	229
Number of edges	2144	2711	4085
+	1303	1691	2206
−	841	1020	1879
+/−	1.55	1.66	1.17
Avg. number of neighbors	19.385	24.188	35.677
Network diameter	9	7	6
Network radius	5	4	4
Characteristic path length	3.020	2.633	2.463
Clustering coefficient	0.486	0.539	0.580
00Network density	0.088	0.108	0.156
Network heterogeneity	0.915	0.641	0.734
Network centralization	0.209	0.189	0.258

## Data Availability

The original contributions presented in this study are included in the article. Further inquiries can be directed to the corresponding author.

## Supplementary Materials

The following supporting information can be downloaded at: https://www.mdpi.com/article/10.3390/ijms27177751/s1.
