# Supplementary material for: The Effect of Mycorrhization with Fungi of Different Efficiency on the Root Metabolome of Medicago lupulina Within Development
Source: Int J Mol Sci. 2026 Aug 29;27(17):7751. doi: 10.3390/ijms27177751 (PMC13565856; doi:10.3390/ijms27177751)
Supplement: Supplementary file 1 [file ijms-27-07751-s001.zip › Supplementary Material Captions.pdf]

Supplementary Materials:

The following supporting information can be downloaded at: https://www.mdpi.com/article/10.3390/ijms27177751/s1.

Figure S1. Productivity and AM activity in *Medicago lupulina* line MlS-1. A. Fresh weight per plant. B. Root fresh weight to shoot fresh weight ratio (RFW:SFW). C. Arbuscule abundance in roots (*A*, %). Different letters ("a" – "g") above bars indicate significant ($p < 0.05$) differences in mean values (ANOVA, Tukey's HSD post hoc test). "+46%" labels above bars in Figure S1.A indicate the mycorrhizal growth response (MGR) calculated per plant weight.

Figure S2. Heatmap of metabolite content in *M. lupulina* roots. Data were normalized by sample median, log-transformed, and standardized. Metabolites were grouped using Pearson correlation and Ward's method. Abbreviations: FFA – free fatty acid; FatAlc – fatty alcohol; na – not annotated; me – methyl ether; RI – retention index; MG – monoacylglycerol. 21 DAS, 35 DAS, 48 DAS, 54 DAS – 21, 35, 48, and 54 days after sowing and inoculation, respectively; "without_AM" – control without AM fungal inoculation; "Ineff_AM" – inoculation with ineffective *Glomus sp.* strain 129.1Te; "Eff_AM" – inoculation with effective *Rhizophagus irregularis* strain RCAM00320.

Figure S3. Analysis of relation between mean number of arbuscules and metabolite profiles. Line plot above importance (increasing MSE after permutation, %) of metabolites in Random Forest Regression. Barplot of factor loadings of predictive component in OPLS model positive loadings correspond to increasing level when number of arbuscules rises. Below line plot of VIP.
